# Supplementary material for: Green Synthesis and Antiproliferative Activity of Gold Nanoparticles of a Controlled Size and Shape Obtained Using Shock Wave Extracts from Amphipterygium adstringens
Source: Bioengineering (Basel). 2023 Mar 30;10(4):437. doi: 10.3390/bioengineering10040437 (PMC10136038; doi:10.3390/bioengineering10040437)
Supplement: Supplementary file 1 [file bioengineering-10-00437-s001.zip › bioengineering-2206067-supplementary.pdf]

Supplementary material

Green synthesis and antiproliferative activity of gold nanoparticles of controlled size and shape obtained using shock wave extracts from *Amphipterygium adstringens*.

SUPPLEMENTARY MATERIAL

Shock waves details

For most applications, these pressure pulses are focused onto a relatively small cigar-shaped volume. The pressure vs. time profile consists of a positive pulse, characterized by a steep onset (rise time t_r between 1 and 500 ns) of up to 150 MPa, followed by a pressure dip with an amplitude that can reach 20 MPa (see the box in **Figure S1a**). The width of the positive ($FWHM^+$) and negative pulse ($FWHM^-$) at half its maximum value equals approximately 0.5 to 3 µs and 2 to 20 µs, respectively.

A method to increase the cavitation energy is to apply a second shock wave that impacts the bubble as it collapses (**Figure S1a**). Because acoustic cavitation involves bubbles with different sizes, the most convenient delay between the two shock waves (i.e., tandem shock waves) is difficult to determine. Moreover, the delay depends on the size of the bubbles and other factors, such as the properties of the fluid, the pressure profile, and the specific goal of shock wave application.

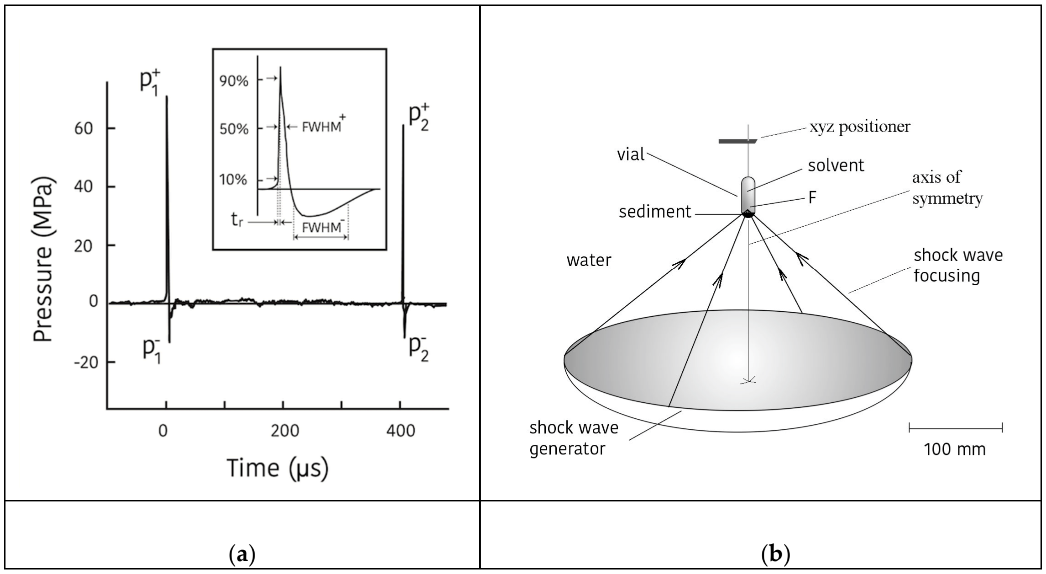

(**a**) (**b**)

Figure S1. **(a)** Pressure profile of two shock waves (tandem "event") emitted at a delay of 400 µs. A pressure vs. time graph at a larger scale appears in the box to show the definition of the rise time (t_r) and the full width at half maximum of the positive ($FWHM^+$) and negative ($FWHM^-$) pulses. **(b)** Sketch of the experimental setup used to expose suspensions of *A. adstringens* inside polyethylene vials to tandem shock waves emitted by a piezoelectric shock wave generator.

Optimization of Ultrasound assisted extraction.

In order to determine the optimal extraction conditions, for the purpose of the study, antioxidant activity was key, since it affects directly in the performance of the extract for the AuNPs synthesis. Variables as power and time were changed to obtain different ultrasound extracts. In **Figure S2** DPPH assay was used to determine antioxidant capacity. The result show that all extracts have an antioxidant capacity lower that the standard used (Trolox). The extracts that were used going forward with the experiments were those which antioxidant capacity was closer to that of the Trolox.

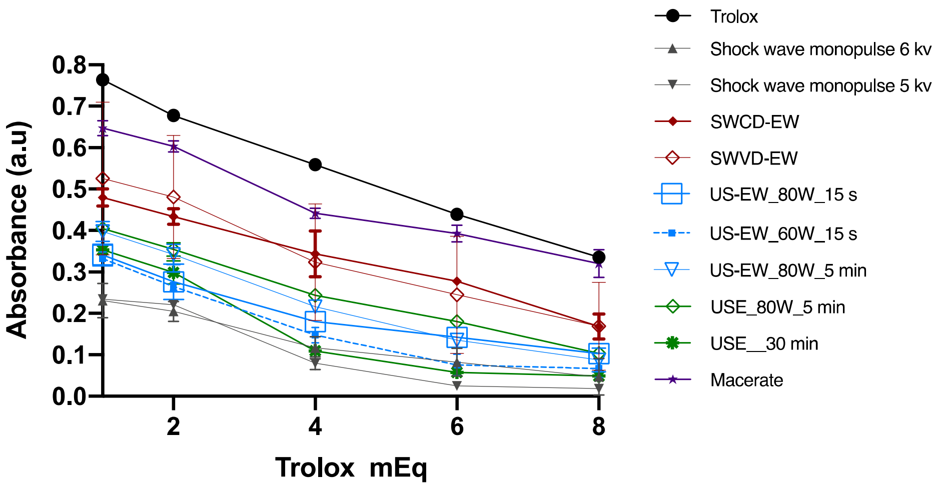

Figure S2. Antioxidant capacity in Trolox mEq of the different extracts obtained by green extraction methods.

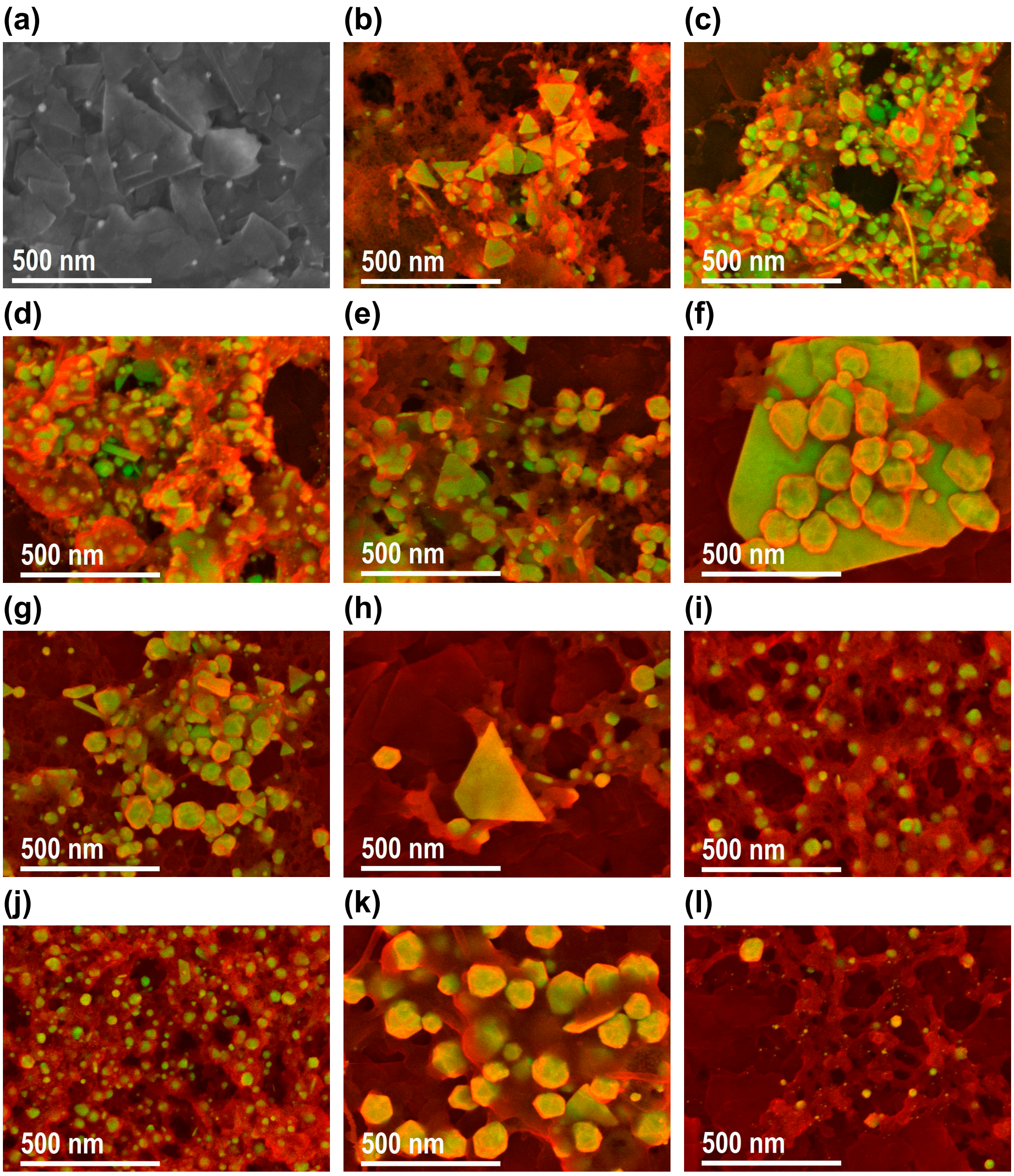

Figure S3. SEM micrographs of AuNPs obtained by green synthesis using different extracts from *A.adstringens*. (**a**) Au@Mac-MeOH, **(b)** Au@US-M, **(c)** u@ SWCD-M, **(d)** Au@ SWVD-M, **(e)** Au@Wm, **(f)** Au@US-W, **(g)** Au@EtWm, **(h)** Au@US-EW, **(i)** Au@SWCD-EW, **(j)** Au@SWVD-EW, **(k)** Au@US-Et, **(l)** Au@SWVD-Et.

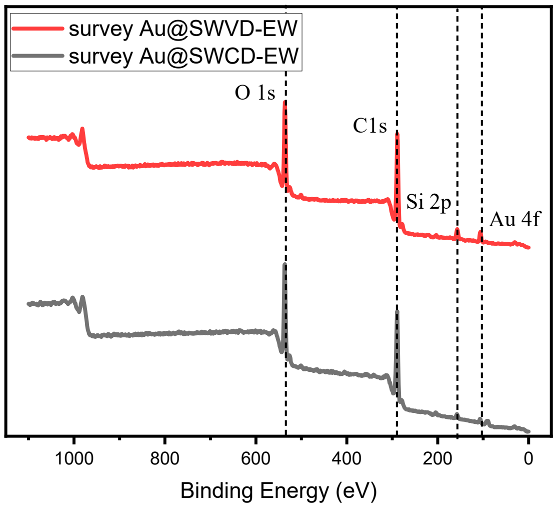

Figure S4. Survey spectra of Au@SWCD-EW and Au@SWVD-EW nanoparticles

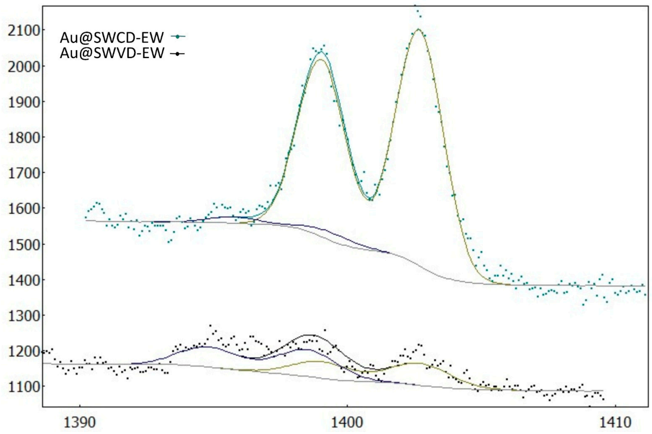

Figure S5. Au 4f high-resolution spectrum of Au@SWCD-EW (green) and Au@SWVD-EW (black) nanoparticles deconvoluted.
